# Short-term changes in air humidity and water availability weakly constrain thermoregulation in a dry-skinned ectotherm

**DOI:** 10.1371/journal.pone.0247514

**Published:** 2021-02-26

**Authors:** Jean-François Le Galliard, David Rozen-Rechels, Anjélica Lecomte, Clémence Demay, Andréaz Dupoué, Sandrine Meylan

**Affiliations:** 1 Sorbonne Université, CNRS, IRD, INRA, Institut d’écologie et des sciences de l’environnement, iEES Paris, UMR 7618, Paris, France; 2 Département de biologie, École normale supérieure, PSL Research University, CNRS, UMS 3194, Centre de recherche en écologie expérimentale et prédictive (CEREEP-Ecotron IleDeFrance), Saint-Pierre-lès-Nemours, France; Universidad de la Republica Uruguay, URUGUAY

## Abstract

Thermoregulation is critical for ectotherms as it allows them to maintain their body temperature close to an optimum for ecological performance. Thermoregulation includes a range of behaviors that aim at regulating body temperature within a range centered around the thermal preference. Thermal preference is typically measured in a thermal gradient in fully-hydrated and post-absorptive animals. Short-term effects of the hydric environment on thermal preferences in such set-ups have been rarely quantified in dry-skinned ectotherms, despite accumulating evidence that dehydration might trade-off with behavioral thermoregulation. Using experiments performed under controlled conditions in climatic chambers, we demonstrate that thermal preferences of a ground-dwelling, actively foraging lizard (*Zootoca vivipara*) are weakly decreased by a daily restriction in free-standing water availability (less than 0.5°C contrast). The influence of air humidity during the day on thermal preferences depends on time of the day and sex of the lizard, and is generally weaker than those of of free-standing water (less than 1°C contrast). This shows that short-term dehydration can influence, albeit weakly, thermal preferences under some circumstances in this species. Environmental humidity conditions are important methodological factors to consider in the analysis of thermal preferences.

## 1. Introduction

Thermoregulation is a critical determinant of the ecological performances of many organisms, and of the sensitivity and resilience of biodiversity to global changes [1–3]. In many ectothermic organisms, thermoregulation largely involves behaviors such as modulations of activity patterns, shifts in the selection of micro-habitats or changes in body posture through which the organism adjusts heat transfer processes to modulate its body temperature [4–6]. A key feature for these organisms is the existence of a modal body temperature that corresponds to the behavioral preference of a sample of individuals at a given time, also called the preferred body temperature [PBT, 7,8]. The PBT is expected to covary with the body temperature that maximizes locomotion, energy gains and demographic performances [reviewed in 9]. The PBT has been measured in a wide diversity of ectotherms ranging from aquatic to terrestrial organisms and from insects to reptiles, and it is usually calculated a single value such as the set-point of body temperature records [9,10]. In dry-skinned terrestrial species, such as lizards, the measurement of PBT is most often carried out under laboratory conditions using thermal gradients or shuttle boxes, minimizing ecological constraints on thermoregulation and standardizing the physiological state of the animals [11 for lizards, reviewed in 12]. Quantification of PBT is particularly useful for predicting activity patterns, life history traits and population dynamics of terrestrial ectotherms [e.g., 13–15]. It is also a crucial step in the construction of mechanistic models to predict their spatial distribution [16].

Studies of thermoregulation in ectotherms have identified numerous factors that generate variation in PBT both within and between species, including seasonal factors, inter-sexual differences, trophic interactions or local environmental conditions [e.g., lizards, 17,18]. In particular, the concept of thermo-hydroregulation proposes that individual water balance and body temperature are jointly regulated through shared physiological and behavioral processes in terrestrial and semi-terrestrial ectotherms [23]. According to this concept, the behavioral needs of hydroregulation could complement or, on the contrary, conflict with the needs of thermoregulation depending on environmental conditions and species-specific features. Studies of wet-skinned ectotherms, such as amphibians, have demonstrated that hydroregulation and thermoregulation are indeed tightly coupled because these species are strongly dependent upon water availability due to their low skin resistance to water loss and reliance on moisture to sustain cutaneous respiration [9,10,19]. In general, wet-skinned ectotherms invest less in thermoregulation behaviors such as basking and are less active thermoregulators than dry-skinned ectotherms [10,20]. Their body temperatures and PBTs are also much more labile and vary importantly with habitat humidity and hydration state [e.g., 21–23]. Dry skinned species can more easily bask to the sun thanks to their skin protection against radiation and higher resistance to evaporative water loss, but the behavioral strategies of these species can also be constrained by water loss risks [24–26]. Yet, we still poorly know the effects of short-term changes in individual water balance on the thermoregulatory behavior of dry-skinned ectotherms, such as lizards and snakes [27,28]. Further studies of the effects of dehydration on thermal preferences in dry-skinned ectotherms are needed to understand their thermo-hydroregulation mechanisms and to reveal how different species of ectotherms respond both to hydric and thermal constraints in their environment [e.g., 29,30].

In reptiles, dehydration is the consequence of an imbalance between water loss, mainly evaporative water loss through the skin and through respiration, and water input, which is accounted for by drinking, at least in snakes and lizards [26,31,32]. Evaporative water losses are determined by the skin resistance to water loss, the intensity of respiratory activity and the microclimatic conditions, particularly the animal’s body temperature and the water vapor deficit between the animal and the surrounding air [26,33]. A chronic dehydration caused by a restriction of drinking water in the laboratory or a lack of free water under natural conditions can compromise thermoregulatory behavior in lizards. For example, prolonged water restriction over several days may lead lizards to select lower body temperatures and reduce their activity [30,34]. Under natural conditions, several reptile species can also modify their activity behavior and their choice of micro-habitats depending on rainfall intensity and availability of free-standing water in the habitat [35–37]. More recently, Sannolo and Carretero [28] further demonstrated that even short-term, acute changes in water availability over a few hours of the day are sufficient to elicit water loss avoidance behaviors to the detriment of thermoregulatory behaviors in four *Podarcis* lizard species. In these species, the changes in thermoregulatory behavior are explained by an increased use of shelters, whose availability in the environment determines opportunities for a behavioral conflict between thermoregulation and hydroregulation [see also, 30]. In a recent parallel study, Pintor et al. [27] also demonstrated that humidity can influence micro-habitat choice during thermoregulation behavior. Yet, to our knowledge, no experimental study has assessed the impact of air relative humidity on thermal preferences. This lack of study may be justified for reptiles because hygrosensation is unambiguously known for insects only and has never been demonstrated in snakes and lizards [19, but see 34],. However, air humidity is a critical environmental determinant of evaporative water loss rates that should influence thermoregulation in most ectotherms and it is therefore important to test its relevance for thermal preference of reptiles.

Here, we tested for short-term effects of daily changes in free water availability and air humidity on the thermoregulatory behavior of a ground-dwelling lizard species, *Zootoca vivipara*, that typical inhabits cold and humid environments across Eurasia. In this species, chronic restriction in water availability leads to individual dehydration and is associated with a decrease in locomotor activity and thermal preferences, and changes in the selection of thermal and moist refuges during the daytime [30,38–40]. In order to study the short-term flexibility of thermoregulation behavior to the risk of dehydration, we measured PBT in thermal gradients installed in climatic chambers maintained under perfectly controlled environmental conditions (temperature, humidity, ventilation, and light). We formulated three hypotheses. First, lizards exposed to water-constrained conditions (low humidity or limited access to free-standing water) are expected to reduce their body temperature in order to decrease their water loss, a phenomenon known as “thermal depression” in acclimation studies [28,34,41]. Second, if this response is the consequence of physiological dehydration during the active time of day rather than a direct behavioral response to an environmental stimulus, the effects of water-constrained conditions are expected to be more pronounced at the end than at the beginning of the day. This is because physiological dehydration is a gradual process that requires hours and days to generate meaningful physiological effects in lizards. Third, if the behavioral response of the animals involves changes in micro-habitat selection like in [28], then the presence of a cold, moist shelter in the thermal gradient allowing for hydroregulation should amplify the changes in thermoregulatory behavior of active lizards.

## 2. Materials and methods

### 2.1. Study site and sample

Experiments were performed under permit 17/DDPP/SPAE/57 to CEREEP-Ecotron and permit 77–01 to J.-F. Le Galliard. We sampled yearling common lizards (*Zootoca vivipara*) from semi-natural populations located at the CEREEP-Ecotron IleDeFrance (48°17’N, 2°41’E) close to the southern distribution of the species range in lowland Northern France. We selected yearlings to standardize age and reduce differences in physiological state (due to reproduction) among individuals. In addition, earlier studies have shown that yearlings have similar average preferences than adults, except pregnant females (Rozen-Rechels et al., in press). Studied individuals were captured between 22–27 May 2017 (May 2017: mean air temperature: 17°C, rainfall: 26.3 mm) from captive populations maintained in fenced, outdoor enclosures (10 x 10 m). In this study site, this sampling period corresponds to the end of the mating season. All yearlings were transferred to a nearby laboratory upon capture, sexed by coloration, morphology and visual search for the presence of hemipenis in males. Lizards were measured for body size (snout to vent length, SVL) and body mass at capture (*n =* 24 males, *n =* 24 females; mean SVL = 53.5 mm ± 3.6 SD, range = 46–62 mm; mean mass = 2.86 g ± 0.7 SD, range = 1.7–4.4 g). Females were longer and weighed higher on average than males (Student’s t tests, all P < 0.001). During maintenance and not experimentation, lizards were individualized in terraria with sterilized peat soil, and kept under stable day-night light and temperature conditions in a temperature-controlled room (16h of night at 16°C and 8h of day at 23°C). Each terrarium provided lizards with a thermal gradient (23 to 35°C) 6h a day (09:00 to 12:00 and 14:00 to 17:00) with a light bulb (25W) at one end of the terrarium. Animals were fed with house crickets three times per week (*Acheta domestica*, 300 ± 10 mg) and provided with water *ad libitum*. We therefore assumed that they were fully hydrated at the start of experimentation and physiologically dependent on water supplementation. Animals were maintained for a minimum of two weeks in captivity before the start of experiments.

### 2.2. Controlled environment facility and experimental design

Measurements of thermal preferences (see 2.3) were conducted in a controlled environment facility allowing precise regulation of air temperature, air humidity, lighting and gas concentrations in the atmosphere [42]. Constant air temperature (20°C), permanent lighting (white light, 2 UVB Reptisun 10.0 neon tube lights suspended above the test cages) and constant gas concentrations (O_2_: 20–21%, CO_2_: 400 ppm) were simulated in two 13 m^3^ climatic chambers during daytime. Air humidity (relative humidity, RH) was adjusted to a constant level that was monitored continuously with a calibrated capacitive sensor (Rotronic HF53/46 HC-S, ± 0.8% RH, ±0.1K at 23°C ± 5°C). Inside each chamber, we installed 12 thermal gradients (80 cm long, 15 cm large and 20 cm deep) warmed at one end with a heat bulb (25 W) located 15 cm above each thermal gradient. Thermal gradients were provided with dry and sterilized peat soil as a substrate, a Petri dish placed at the center of the box to manipulate water availability, and a wood plate on the warm side of the box to enhance basking efficiency. We recorded the range of substrate temperatures from the cold to the warm range of the box before and at the end of each daily trial. Extreme temperatures ranged from 22–25°C on the cold side to 45–50°C on the warm side, thus allowing behavioral selection of optimal body temperatures for common lizards [between 25–40°C during field activity, 43].

Animals were first tested for their thermoregulation behavior inside the climate chamber in a pilot study (see below), which further allowed them to familiarize with the set up. Then, we designed three independent experiments performed with the same sample of lizards. In order to evaluate the hypothesis that lizards exposed to acute restrictions on water availability are expected to reduce body temperature, we manipulated air relative humidity and free water availability in the thermal gradient (Experiment 1). During this first experiment, performed between June 9 and 16, we contrasted a “dry treatment”:(20% RH) with a “wet treatment” (80% RH) and further manipulated at the same time free water availability in the thermal gradient (Petri dish filled with water or empty during the day). Environmental conditions were thus equivalent to a water vapor pressure deficit (VPD, calculated with Magnus equation) of 1.87 kPa and 0.46 kPa at 20°C in the dry and wet treatments, respectively. All animals were tested four times sequentially with just one daily trial per treatment condition, the sequence of four treatment conditions being pseudo-randomly determined to avoid confounding treatment effects with trial number. Thus, lizards were observed for 1 full day in each treatment condition. Lizards were fed right after each trial and maintained in the laboratory during a minimum rest period of 2 days without food but with *ad libitum* access to water between each trial. Fasting ensured that most animals were post-absorptive during the tests [44], but we cannot entirely exclude that some defecated small amount of excreta.

In order to evaluate the hypothesis that lizards exposed to a milder change in air humidity also reduce their body temperature, we manipulated air relative humidity in a moister range of atmospheric conditions (Experiment 2). During this second experiment, performed between June 28 and July 1st, we compared dry and wet conditions ranging from 70% RH (“wet treatment”) to 95% RH (“super wet treatment”, equivalent to VPD of 0.70 kPa and 0.12 kPa at 20°C, respectively). This range of moisture is probably more relevant to the natural variability seen across micro-habitats exploited by this species inside natural populations [38]. Lizards were not provided with free-standing water during this experiment. All animals were tested two times sequentially with one daily trial per treatment condition, the sequence of two treatment conditions being pseudo-randomly determined to avoid confounding treatment effects with trial number. Lizards were fed right after each trial and maintained in the laboratory during a minimum rest period of 2 days without food but with *ad libitum* access to water between each trial.

In order to evaluate the hypothesis that micro-habitat selection plays an important role in the thermoregulatory behavior of active lizards, we repeated the second experiment with a set-up where lizards could use a cold, moist shelter in the thermal gradient (Experiment 3). During this third experiment, performed between July 5 and July 9, we enriched the thermal gradient with a shelter (made out of a piece of cardboard, 15 cm long and 15 cm large) located above the substrate on the cold side of the thermal gradient. Soil below the shelter was made fully wet at the start of each trial to create a cold and wet microhabitat during the day (mean temperature of 20–21°C, air humidity of 90–100%, iButton Hygrochron DS1923-F5). When lizards were inside the shelter, we did not disturb them and did not record their body temperature. We manipulated air relative humidity in the same way than in the second experiment (wet treatment”: 70% RH, “super wet treatment”: 95% RH, equivalent to VPD of 0.70 kPa and 0.12 kPa at 20°C, respectively) and did not provide free-standing water during experimentation. All animals were tested two times sequentially with one daily trial per treatment condition, the sequence of two treatment conditions being pseudo-randomly determined to avoid confounding treatment effects with trial number.

### 2.3. Measurements of thermoregulatory behavior

Post-absorptive lizards kept without food for 2 days in the laboratory prior to each trial were removed from their home cage, measured for body mass at 8:00 am and then placed alone in each gradient at least 1:30 hours prior to the start of observations. This procedure was chosen to eliminate effects of digestion on body temperatures and to decrease the effects of handling stress [45]. We acknowledge that acclimation time was shorter than in some similar studies with lizards because lizards were not exposed to the gradients at least 24h before testing and therefore some of the results may be confounded with exploratory behavior. However, we are confident that the acclimation time was long enough to eliminate confounding effects of handling stress and shyness. In order to quantify if thermoregulatory responses are the consequence of physiological dehydration during the day rather than a direct behavioral response to an environmental stimulus, the same observer then recorded the body temperature of each lizard every 40 minutes from 10:00 am to 5:20 pm. This corresponds to the standard activity period of common lizards. Animals were weighed at the end of each trial around 5:30 pm and returned to their home cage. Differences in body mass during the day were then calculated to quantify daily mass loss without food intake. Mass loss in common lizards includes multiple components but is prominently due to cutaneous and respiratory water loss under these experimental conditions [38]. The common lizard is a cool-climate, mesic species with low cutaneous resistance to water loss relative to other squamate reptile species [25].

Surface body temperature was measured with a calibrated infrared thermometer (Raytek, Raynger MX2) at ca. 20–30 cm from the target. Cloacal body temperatures measured with a K-type thermocouple are highly correlated with these surface skin temperatures [46]. Similar to previous studies of squamate reptiles, we considered these measures as best estimates of behaviorally selected body temperatures during the activity period of the day in the absence of thermoregulation costs [14,47]. Preferred body temperature (PBT) was calculated as the daily average of body temperatures [48], thermal precision was calculated as the daily standard deviation of body temperatures (low values indicate more precise thermoregulation), and set-point range was calculated from the central 50% of all body temperatures [49]. In addition, we ran a qualitative, pilot study with one climatic chamber where 60 lizards were observed to detect any obvious disturbance caused by the contained environment (noise, electromagnetic disturbance, etc.) of the climatic chamber. We found in this pilot study that the T_b_ of lizards ranged from 25 to 35°C during the day and matched qualitatively the values recorded in earlier studies (mean = 31.75 ± 0.04 SE, 50% T_b_ breadth [30.11, 33.7]) using laboratory thermal gradients or performed in outdoor conditions [47,50,51]. In addition, we found that T_b_ tended to decrease during the day and lizards were predominantly observed basking (53% of records) and in the warm side of the box (41% of records), especially at the beginning of the day. This parallels qualitatively the thermoregulatory behavior seen in a previous laboratory study [46], which indicated that the set-up did not overly stress the lizards.

### 2.4. Statistical analyses

All statistical analyses were done using the software R version 3.4.3 and the *nlme* package for mixed model fitting [52]. We fitted statistical models separately to data from each experiment even though they were run with the same animals since we were primarily interested in testing the effects of environmental conditions on thermoregulation behavior. Given that experiments had to be run in sequence, we acknowledge that differences between values of each experiment might represent genuine effects of time passing and acclimation to experimentation.

First, we calculated for each individual and each daily trial the PBT, the SD of PBT (an index of thermal precision) and the body mass change (difference in body mass between the end of the start of each trial, our proxy of daily water loss). The PBT was calculated as the mean T_b_ of each daily trial and its SD was calculated from the 12 data points of each daily trial to provide summary statistics for the thermal preferences and the precision of thermoregulation. We then analyzed the variation of each of these three variables with a linear mixed model accounting for repeated measurements on the same lizards over consecutive trials. In the full linear mixed models, we included the main fixed effects of sex, treatment groups and their interactions to take into account the factorial ANOVA design. We further included individual identity as a random intercept factor. These analyses were based on 192 records in Experiment 1 and 96 records records in Experiments 2 and 3.We did these analyses because this is the classical approach to quantify thermal preferences statistics in lizards.

Second, we independently used repeated measurements of T_b_ from each individual and daily trial to characterize better intra-individual variation in body temperature during the daytime. We analyzed T_b_ with a linear mixed model including fixed effects of sex, treatment groups and time of the day and their interactions to take into account the factorial ANCOVA design. The covariate time of the day was calculated as the number of minutes since the start of the experiment at 10:00 am and was centered prior to analysis to ease interpretation of parameter estimates. Time of the day might represent a dehydration and/or starvation effect or some intrinsic diurnal variation in thermal preferences of lizards. We used a linear rather than a curved relationship with time of the day based on graphical explorations of the data. We further included individual identity as a random intercept factor. These analyses were based on 2320 records of body temperature in Experiment 1, 1138 records in Experiment 2 and 776 records in Experiment 3.We did these analyses because this allowed us to test the hypothesis that treatment influenced daily changes of body temperature instead of mean body temperature.

All linear mixed models for PBT, SD of PBT, body mass change and T_b_ were fitted using the *lme* function. The T_b_ data were transformed with a Box-Cox power transformation to ensure the normality and homogeneous variance of the residuals of the model [53]. Model selection was done by backward elimination of non-significant factors according to marginal F statistics, where the denominator degrees of freedom are determined by the grouping level (inner-outer method) at which the term is estimated [52]. This procedure is similar to type III sums of squares of classical ANOVA. Post-hoc tests of differences among groups were performed using the Tukey-Kramer method for unequal sample size. All results are reported as means ± SE unless otherwise stated.

## 3. Results

### 3.1. Summary daily statistics of thermoregulation

**Fig 1:**
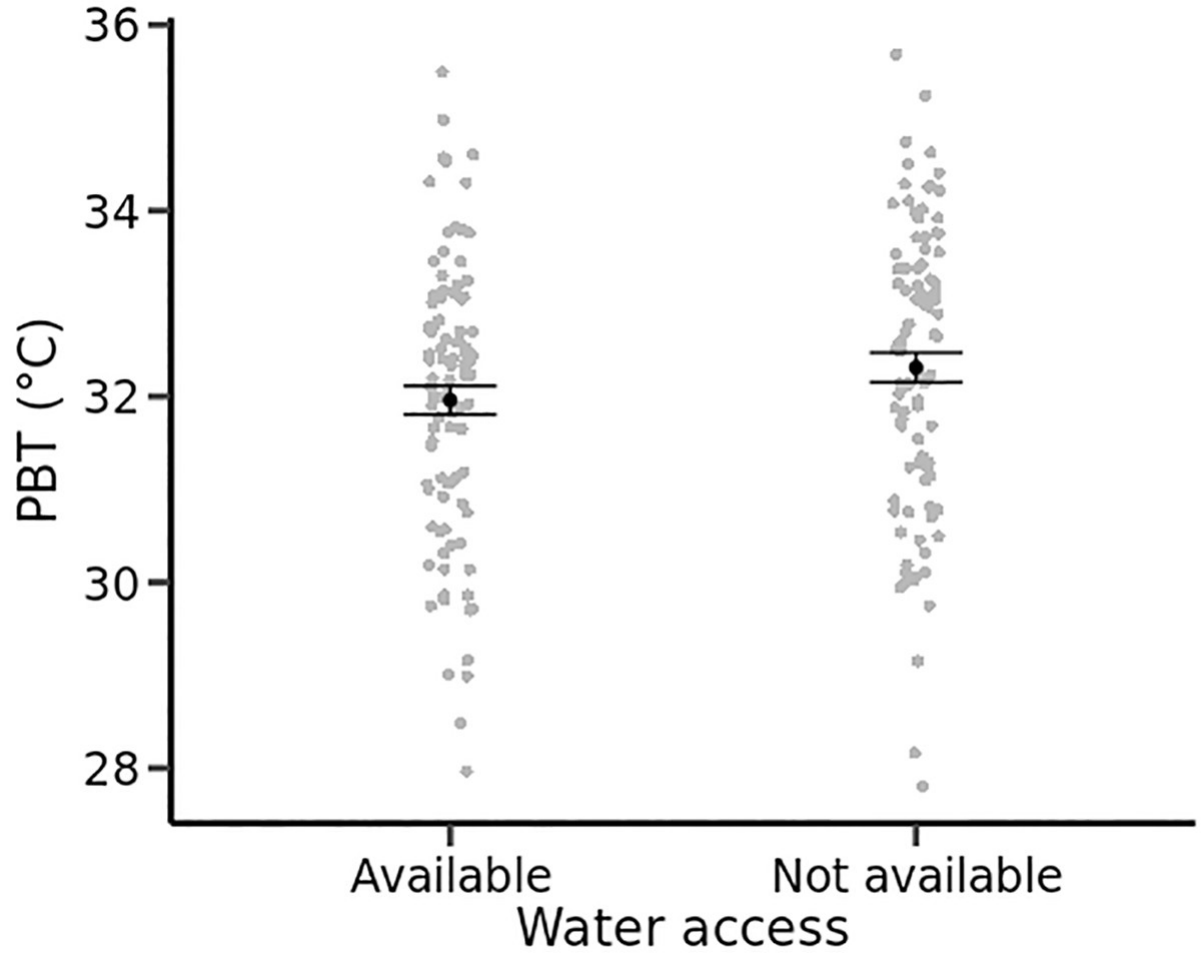
Preferred body temperatures (mean daily body temperatures, °C) of yearling common lizards from thermal gradients are slightly higher with than without access to drinking water. Mean, standard errors and dotplots are obtained from combined raw data of males and females.

In Experiment 1, PBT was higher in males than in females (F_1,46_ = 6.74, P = 0.01) and when free water was available than in water-restricted conditions (F_1,142_ = 4.28, P = 0.04, Fig 1). The change in thermal preferences between water availability treatment groups was not a consequence of lizards simply avoiding the warmest parts of the gradients where the expected evaporation rates would be the highest (Fig 1). The SD of PBT was jointly influenced by sex (F_1,46_ = 9.39, P = 0.004) and a two-way interaction between water availability and air humidity (F_1,141_ = 4.21, P = 0.04). The SD of PBT was higher when air humidity was high and when free water was not provided (Table 1). In Experiments 2 and 3, daily statistics of thermoregulation were not influenced by treatments but differed between sexes (all P < 0.05 for sex differences): males had higher PBT and a higher SD of PBT than females (Table 1).

**Table 1:** Descriptive statistics for thermal preferences including preferred body temperatures (PBT), standard deviation around the mean (an index of thermal precision), and set-points (50% quantiles, included for meta-analyses) in males and females from each treatment.

	PBT		SD of PBT		Set-points	
	Females	Males	Females	Males	Females	Males
**Experiment 1—manipulation of water availability and air humidity in neutral arenas**						
Water not available—dry	31.19	32.22	2.3	2.76	[29.6, 32.8]	[30.5, 34.2]
Water not available—wet	31.98	32.43	1.79	2.39	[30.8, 33.2]	[31.0, 33.9]
Water available—dry	32.25	32.74	1.92	2.82	[31.3, 33.4]	[31.4, 34.6]
Water available—wet	31.61	32.68	2.19	2.83	[30.5, 33.0]	[30.8, 34.5]
**Experiment 2—manipulation of air humidity in neutral arenas**						
Wet	30.54	32.97	1.67	2.59	[29.57, 31.7]	[31.0, 34.8]
Super wet	30.75	32.95	1.62	2.47	[29.71, 31.7]	[31.3, 34.6]
**Experiment 3—manipulation of air humidity in neutral arenas with a shelter**						
Wet	30.47	33.78	1.61	2.05	[29.5, 31.3]	[32.8, 34.9]
Super wet	30.74	33.61	1.57	1.73	[30.1, 31.6]	[32.8, 34.6]

Note that the differences between values of each experiment might represent genuine effects of time passing and acclimation to experimentation.

### 3.2. Daily pattern of thermoregulation in Experiment 1

**Fig 2:**
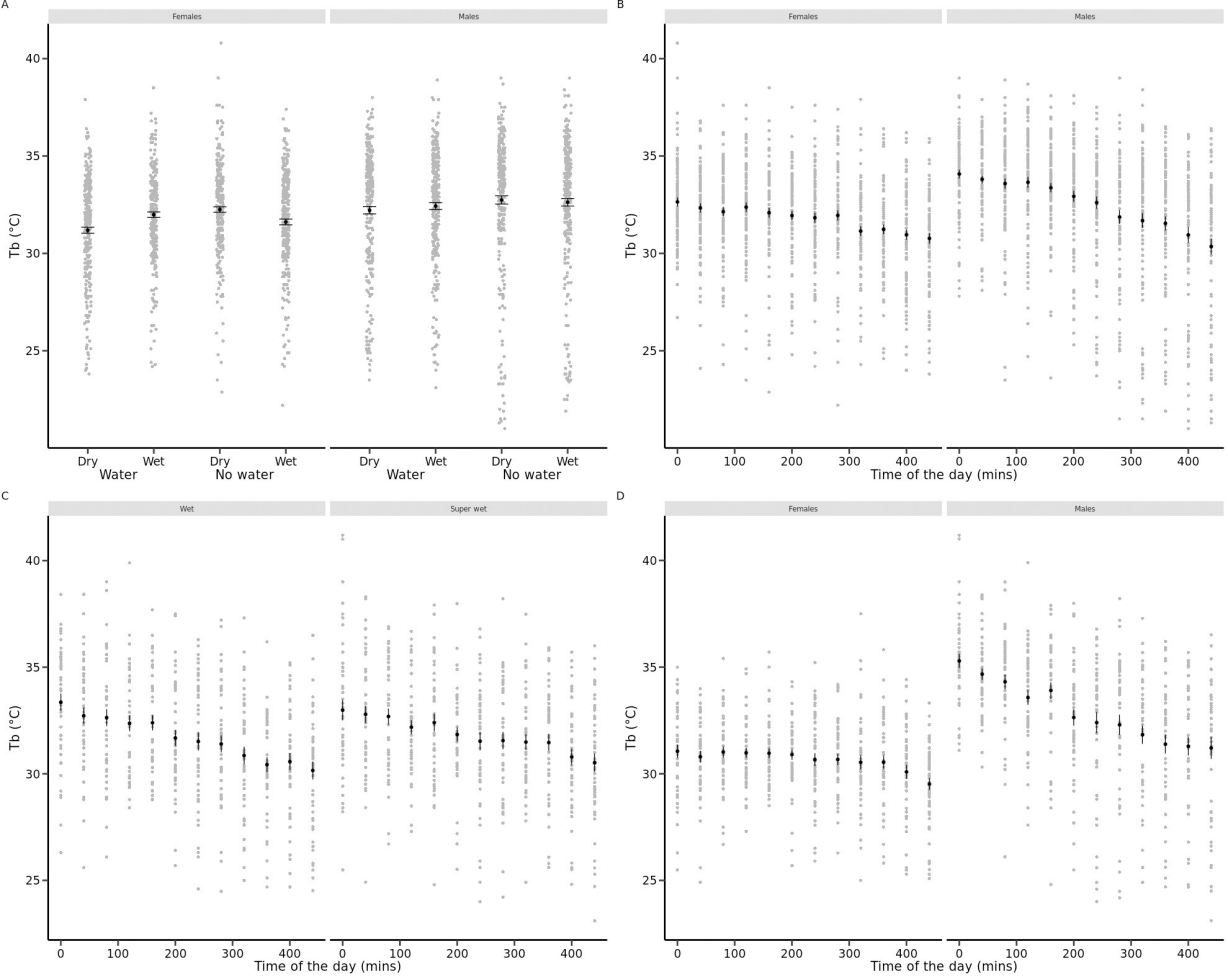
Individual records of body temperatures (T_b_, °C) of yearling common lizards during daytime. A. Mean, standard errors and dotplots for female and male lizards in neutral arenas from Experiment 1 as a function of availability of free-standing water and air humidity (dry: VPD = 10.66 kPa, wet: VPD = 0.46 kPa). B. Mean, standard errors and dotplots for female and male lizards in neutral arenas from Experiment 1 per daytime period. C. Mean, standard errors and dotplots for lizards of both sexes in neutral arenas from Experiment 2 per daytime period in each air humidity treatment (wet: VPD = 0.70 kPa, super wet: VPD = 0.12 kPa). D. Mean, standard errors and dotplots for female and male lizards in neutral arenas from Experiment 2 per daytime period.

**Fig 3:**
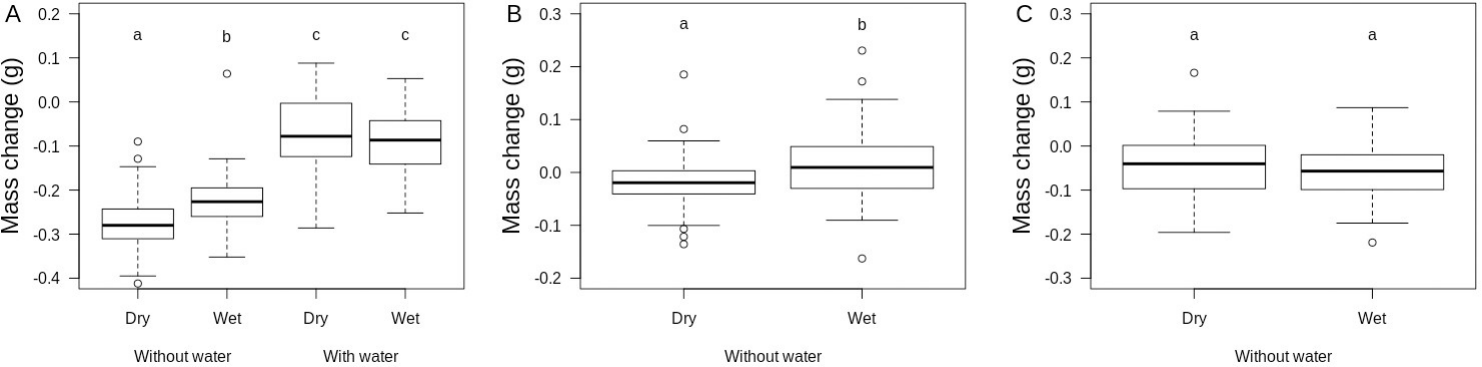
Boxplots of daily body mass change of yearling common lizards in three independent experiments. A. Effects of water availability (thermal gradient with or without water) and air humidity (dry: VPD = 1.87 kPa, wet: VPD = 0.46 kPa) in neutral arenas from Experiment 1. B. Effects of air humidity (wet: VPD = 0.70 kPa, super wet: VPD = 0.12 kPa) in neutral arenas from Experiment 2. Data are residual body mass change after controlling for sex differences and initial body mass. C. Effects of air humidity (same as B) in neutral arenas provided with shelters from Experiment 3. Letters indicate statistically different groups according to contrasts from the statistical models explained in the main text. Note that differences in mean mass loss between experiments are difficult to interpret because those were performed sequentially and lizards might acclimate to laboratory conditions.

In Experiment 1, animals were tested under very low (RH = 20%) against high (RH = 80%) humidity conditions in the presence or in the absence of free water. A complex three-way interaction model between sex, air humidity treatment and water availability best explained daily variation in T_b_ (Table 2). In females, body temperature was higher on average when free water was available (post-choc contrast of intercept = +0.33°C ± 0.13, P = 0.01, Fig 2A) and it decreased with time of the day (-0.006°C.min-^1^ ± 0.0008, P < 0.0001, Fig 2B) but proportionally less when free water was available (post-choc contrast of slope = +0.0017°C.min-^1^ ± 0.0009, P = 0.06; equivalent to 0.8°C contrast at the end of the day) and when air humidity was wet (post-choc contrast of slope = +0.002°C.min-^1^ ± 0.0009, P = 0.04; equivalent to 0.9°C contrast at the end of the day). In males, body temperature was also higher on average when free water was available (post-choc contrast of intercept = +0.35°C ± 0.13, P = 0.01) and it decreased with time of the day (-0.008°C.min-^1^ ± 0.0006, P < 0.0001) independently from free water availability and air humidity. Animals lost on average 0.16 g (± 0.11 SD) during the experiment. Body mass loss was significantly influenced by a two-way interaction between air humidity and water availability (F_1,184_ = 5.3, P = 0.02) after controlling for differences between sexes. Availability of free water canceled out body mass loss as well as the negative effect of air dryness on body mass change (see Fig 3A).

**Table 2:** Best selected models for intra-individual variation in body temperature (°C) in experiments 1 to 3.

	F statistics	Parameter estimates
**Experiment 1—manipulation of water availability and air humidity in neutral arenas without a shelter**		
Intercept		32.09 ± 0.29
Time of the day (mins)	F_1,2261_ = 51.45, P < 0.0001	-0.005 ± 0.0006
Air humidity (80% versus 20%)	F_1,2261_ = 3.9, P = 0.05	0.43 ± 0.22
Water availability (with versus without)	F_1,2261_ = 9.97, P = 0.002	0.70 ± 0.22
Sex (males versus females)	F_1,46_ = 10.02, P = 0.003	1.07 ± 0.34
Time of the day × Water availability	F_1,2261_ = 2.4, P = 0.12	0.001 ± 0.0008
Time of the day × Sex	F_1,2261_ = 29.37, P < 0.0001	-0.004 ± 0.0008
Air humidity × Water availability	F_1,2261_ = 4.91, P = 0.03	-0.71 ± 0.32
Air humidity × Sex	F_1,2261_ = 4.22, P = 0.04	-0.62 ± 0.30
Water availability × Sex	F_1,2261_ = 3.62, P = 0.06	-0.58 ± 0.30
Air humidity × Water availability × Sex	F_1,2261_ = 7.45, P = 0.006	1.18 ± 0.43
**Experiment 2—manipulation of air humidity in neutral arenas without a shelter**		
Intercept		31.08 ± 0.20
Time of the day (mins)	F_1,1085_ = 63.58, P < 0.0001	-0.003 ± 0.0008
Air humidity (95% versus 70%)	F_1,1085_ = 1.67, P = 0.19	0.17 ± 0.13
Sex (males versus females)	F_1,46_ = 77.35, P < 0.0001	2.25 ± 0.25
Time of the day x Sex	F_1,1085_ = 53.72, P < 0.0001	-0.007 ± 0.0009
Time of the day x Air humidity	F_1,1085_ = 3.18, P = 0.07	0.0017 ± 0.0009
**Experiment 3—manipulation of air humidity in neutral arenas with a shelter**		
Intercept		30.67 ± 0.24
Time of the day (mins)	F_1,723_ = 0.17, P = 0.68	-0.0003 ± 0.0007
Air humidity (95% versus 70%)	F_1,723_ = 3.71, P = 0.05	0.38 ± 0.20
Sex (males versus females)	F_1,46_ = 91.36, P < 0.0001	3.35 ± 0.33
Time of the day x Sex	F_1,723_ = 5.31, P = 0.02	-0.002 ± 0.001
Sex x Air humidity	F_1,723_ = 7.63, P = 0.006	-0.80 ± 0.29

Reference groups are females, lizards without access to free water and the driest humidity group.

### 3.3. Daily pattern of thermoregulation in Experiment 2

In Experiment 2, animals were tested against medium (RH = 70%) and very high (RH = 95%) humidity conditions without access to free-standing water. The best model for intra-individual variation in T_b_ included a two-way interaction between air humidity treatment and time of the day and a two-way interaction between sex and time of the day (Table 2). On average, body temperatures were higher in males than in females and decreased with time of the day, especially in males (Fig 2D). The decrease of T_b_ during the day was marginally more pronounced in the wet conditions (Fig 2C). However, this effect of air humidity was small, equivalent to a 0.3–0.4°C decrease in T_b_ at the end of the day in dry relative to wet conditions. Animals lost on average 0.18 g (± 0.07 SD) during the experiment, and body mass loss was higher in dry than in wet conditions (F_1,45_ = 14.5, P = 0.0004) after correcting for effects of sex (F_1,46_ = 15.4, P = 0.0003) and initial body mass (F_1,46_ = 5.76, P = 0.02, see Fig 3B).

### 3.4. Daily pattern of thermoregulation in Experiment 3

In Experiment 3, animals were tested against medium (RH = 70%) and very high (RH = 95%) humidity conditions without free-standing water but in the presence of a wet and cold shelter. The best model for intra-individual variation in T_b_ included a two-way interaction between air humidity treatment and sex and a two-way interaction between sex and time of the day (Table 2). In wet conditions, males had marginally lower T_b_ (post-choc contrast of intercept = -0.41°C ± 0.23, P = 0.07) while females had higher T_b_ (post-choc contrast of intercept = +0.39°C ± 0.18, P = 0.04) than in dry conditions. In addition, a decrease of T_b_ during the day was seen in males (-0.003°C.min-^1^ ± 0.0008, P = 0.0009) but not in females (-0.0003°C.min-^1^ ± 0.0007, P = 0.65). Animals lost on average 0.05 g (± 0.07 SD) during the experiment, but body mass loss was not influenced by air humidity (F_1,44_ = 1.23, P = 0.27) nor by sex (F_1,46_ = 1.07, P = 0.31, see Fig 3B).

## 4. Discussion

Taking into account the water balance and its interaction with thermoregulation is crucial because the hydration status of an animal is expected to modify its thermoregulatory behavior, even in dry-skinned ectothermic species more resistant to water loss [27,54]. We performed a series of three experiments with the same animals to assess the effects of water conditions on thermal preferences in a controlled environment where air humidity was stable and constant during the day. Although our study design had some limitations, such as the artificial environment, short acclimation time of lizard inside neutral arenas and non-independence of the three repeated experiments, it suggests that short-term changes in hydration can influence, albeit weakly, the lizard’s thermoregulation under some circumstances. First, our experimental study confirms that the dehydration status of a lizard during the day, assessed by a greater body mass loss [55], depends on the availability of free-standing water and on atmospheric humidity. Second, we found that short-term dehydration leads to subtle but significant changes in thermoregulatory behaviors. Faster dehydration caused by a restriction in water availability is associated with lower body temperatures on average, particularly at the end of the day and in females, but also with a more precise thermoregulation. This effect was however small with a short-term thermal depression of less than 0.5°C on average. Faster dehydration caused by a reduction in atmospheric humidity subtly influences daily thermoregulation patterns: in dry conditions, some lizards have slightly lower body temperatures at the end of the day. This effect was also relatively small with a thermal depression of less than 1°C on average at the end of the day. The presence of a cool, moist shelter in the thermal gradient did not amplify this effect. These results suggest a potential trade-off between water balance regulation and the needs of thermoregulation at high body temperatures, although changes of thermal preference were relatively minor and may have a little short-term effects on performance of lizards given that this lizard species is a thermal generalist with a relatively wide thermal performance breadth [46,56].

The mean daily thermal preference of common lizards changed significantly in the absence of free-standing water in the thermal gradient but was not influenced by atmospheric humidity even when we compared an extremely dry and wet air. In the absence of drinking water, the lizards lost significantly more body mass during the day, in the order of 7–10% of their initial body mass depending on the ambient humidity. The preferred body temperature, calculated as the daily average, decreased significantly by about 0.5 to 1°C and the statistical dispersion of body temperatures tended to narrow around this lower average in the absence of drinking water. These effects of free water availability on thermal preference are weak but qualitatively similar to those observed in the lizard *Sceloporus undulatus* [34] and in four different species of lizards of the genus *Podarcis* [28] as well as in the fossorial legless lizard *Anniella pulchra* [57]. These differences in PBT may reflect a behavioral strategy to reduce water loss independently of changes in activity since our measurements are made in a thermal gradient without a shelter. In particular, analyses of thermoregulation behavior show that animals use the warm end of the thermal gradient more often in the presence of free-standing water, but produce the same thermoregulation effort (percentage of lizards performing basking behaviors, data not presented). The potential benefit of lowering body temperature to reduce evaporative or respiratory water loss must be accompanied by a small reduction in the animals’ performance since body temperatures shifted slightly away from the optimum temperature for locomotion, food intake and body growth. However, given that the thermal performance breadth is relatively wide in the common lizard, the reduction in performance is likely to be very small and of little biological consequence relative to daily variation induced by other factors such as air temperature or fear of predation [46,56,58].

Quantitatively, the decrease in body temperature observed in this study remains relatively small compared to that observed (2–4°C) in four species of *Podarcis* lizards by Sannolo and Carretero [28]. This result is surprising and was not expected *a priori* because the common lizard is a species less resistant to water loss than *Podarcis* lizards [25,38,59]. It is difficult to fully explain these differences since experimental protocols of the two studies are not identical, but one possibility is that common lizards also adjusted their locomotor activity in the thermal gradient by moving less often in drier conditions. The common lizard is an actively foraging species compared to the more sit-and-wait foragers such as *Podarcis* lizards and this foraging mode should be sensitive to water restriction, with lizard moving less and saving more water by reduced movement effort in more desiccating environments [60,61]. Since lizards of the genus *Podarcis* are species with a more sit-and-wait foraging mode, they might have less opportunities to adjust their behavioral activity and their “only” behavioral solution is therefore to lower importantly their body temperature in the absence of water. Unfortunately, we did not record movement activity in this study but previous investigations with lizards have shown that dehydration can reduce locomotor activity [29,43]. These hypotheses will have to be tested by studying a greater number of contrasting species with distinct foraging styles in the future and recording both body temperature and movement activity.

The analysis of daily thermoregulation patterns reveals subtler effects of water conditions. In general, lizards bask at higher temperatures at the beginning than at the end of the day, as highlighted in our previous work using the same protocol with a thermal gradient [40,46] or with more complex experimental arenas [30]. The effects of free-standing water availability are more pronounced at the end of the day than at the beginning: in the presence of free water, lizards maintain a higher preferred temperature that is more stable over time during the day, whereas a decrease in body temperatures is observed at the end of the day under water restriction conditions, especially in females (effect size of ca. 0.8°C). A similar time-dependent effect is observed for dry versus wet atmospheric humidity: a decrease in body temperatures is observed at the end of the day in dry compared to wet conditions, especially in females (effect size of ca. 0.9°C). The same result was not found when we compared wet and super-wet atmospheric conditions, probably because contrasts in air humidity were too weak to induce detectable changes in hydration state and thermoregulation behaviors on the short-term. In this way, it is predicted that the most dehydrating conditions should alter natural hourly pattern of activity when thermal conditions fluctuate during the day [29,39]. In particular, common lizards exposed to strongly dehydrating conditions are expected to reduce their activity more importantly during heat waves and especially at the end of the day [62].

The daily patterns of thermoregulation are broadly consistent with the hypothesis that thermoregulatory behavior adjusts to individual variation in hydration status rather than to environmental stimuli concerning the presence of water (via vision, for example) or air humidity [via hygrosensation, 63]. Indeed, if flexible changes in thermoregulatory behaviors were an anticipated response to the perceived risk of dehydration, a uniform response should be observed throughout the day and especially at the beginning of the day. The fact that the response is primarily observed at the end of the day is instead consistent with a behavioral response to the linear decrease in body mass and stronger individual dehydration state at the end of the day. Furthermore, this hypothesis also explains the fact that the behavioral response is weaker for air humidity, since differences in air humidity had much smaller effects on individual mass loss (and therefore water balance) than daily water restriction. It also explains why overall effects of our manipulation on thermal preference were generally small since experiments lasted only one day, an insufficient time to induce a severe dehydration. Other studies have shown that changes in thermoregulation and activity were proportional to the dehydration status of individuals in several species of reptiles [29,30,34]. In contrast, Sannolo and Carretero [28] observed short-term behavioral responses to water restriction suggesting that *Podarcis* lizards might be sensitive to the perception of dehydration risk. Indeed, in these species, adults exposed to water restriction adopt more cautious thermoregulatory behaviors from the beginning of the day, at a time when they are very unlikely to be physiologically dehydrated. We suggest that more studies should be performed to understand the behavioral rules and threshold functions dictating the behavioral conflicts between water balance and body temperature regulation in lizards [e.g., 64].

The thermoregulatory behaviors are modified by the presence of a cold, damp shelter that limits water losses in drying conditions and allows lizards to reduce their thermoregulation behavioral activity. Lizards used the wet refuge ca. 30% of the time during the day. However, the observed changes in thermoregulatory behaviors are not consistent with our hypothesis that the presence of a cold and wet shelter should amplify the trade-off between hydroregulation and thermoregulation. First, we found that more desiccating conditions were not associated with higher lizard mass losses when we provided lizards with a shelter contrary to the two other experiments. This suggests that the use of shelter allows lizards to buffer effects of air humidity on the water balance. Second, we found a lower body temperature of females under the most dehydrating conditions, which was of the same order of magnitude as the response to complete water deprivation in Experiment 1. Surprisingly, however, the opposite result is observed in males. One possibility for interpreting these results could be sex differences in the prioritization of shelter use versus active thermoregulation, perhaps also in response to the repeated stress caused by the presence of an experimenter to measure body temperatures. For example, males might prioritize strategies to reduce water loss by using more shelters in drier conditions and this could allow them to capitalize on activity phases to increase their body temperature. These explanations remain very speculative insofar as the frequency of shelter use does not seem to be related to the treatment conditions in this experiment (data not presented), but they confirm earlier observations of sex-specific responses to water restriction in this species [30,65].

## 5. Conclusions

Natural populations of ectotherms are exposed to joint changes in water and thermal conditions, the consequences of which can only be predicted with studies that integrate the interactions between these two factors [26,36]. In reptiles, it has been suggested that rapid, daily changes in the water status of individuals can generate flexible, equally rapid changes in thermoregulatory behavior [28,34,57]. Our results confirm these previous observations, indicate that the thermal preferences of common lizards are sensitive to changes in free-standing water and air humidity, and suggest that these flexible changes are driven by individual hydration state. However, these effects were generally weak (less than 1°C on average) and thus unlikely to influence dramatically locomotor or foraging performances of the lizards on the short-term. These results add to a growing list of studies indicating that hydroregulation and thermoregulatory behaviors cannot be understood separately from each other and may often trade-off in dry-skinned ectotherms [54]. On a methodological ground, this also strengthens the fact that environmental humidity conditions are important methodological factors to consider in the analysis of preferred temperature variation patterns.

## Abbreviations

PBT: preferred body temperature
T_b_: body temperature
RH: relative humidity
VPD: water vapor pressure deficit
